# Development of a resource-use measure to capture costs of diabetic foot ulcers to the United Kingdom National Health Service, patients and society

**DOI:** 10.1177/17449871231208108

**Published:** 2023-12-27

**Authors:** Katherine Cullen, Mari Jones, Christina Sheehan, Frances Game, Kavita Vedhara, Deborah Fitzsimmons

**Affiliations:** Research Officer, Swansea Centre for Health Economics, Swansea University, Swansea, UK; Research Officer, Swansea Centre for Health Economics, Swansea University, Swansea, UK; Programme Manager/Research Support Officer, School of Medicine, University of Nottingham, Nottingham, UK; Consultant and Director, Royal Derby Hospital, University Hospitals of Derby and Burton NHS FT, Derby, UK; Professor, Centre for Academic Primary Care, University of Nottingham, Nottingham, UK; School of Psychology, Cardiff University, Cardiff, UK; Professor, Swansea Centre for Health Economics, Swansea University, Swansea, UK

**Keywords:** adults, diabetes, instrument development, methods, public and patient involvement, statistical analysis, survey

## Abstract

**Background:**

Diabetic foot ulcers (DFUs) add a significant burden to the lives of people with diabetes in the United Kingdom. They can have a considerable impact on a patient’s daily life, with treatment requiring frequent changes of dressings and clinic attendances. Nurses and other allied health professionals (AHPs) within the community provide most wound care representing the primary cost driver.

**Aims:**

To collaboratively explore key resource use related to the management of DFUs to develop, and pilot, a participant-reported measure to inform economic evaluations.

**Methods:**

A literature search and semi-structured interviews determined health and non-health resource use in management of DFUs. A consensus view of the selected items was established in a modified Delphi study and further tested for acceptability and validity in a pilot study.

**Results:**

Primary care consultations with a podiatrist or orthotist, district nurse visits, out-of-hours and emergency care, scans and investigations, and consumables provided in clinics were rated as the most important resource use items.

**Conclusions:**

This work has informed the development of a measure that captures resource use considered important by the people most affected by DFUs; patients, family members and carers, and the healthcare professionals key to DFU management.

## Introduction

Diabetic foot ulcers (DFUs) are a significant problem affecting between 1% and 2% of people with diabetes annually (Chamberlain et al., 2022; Kerr et al., 2019; NHS Digital, 2019). Management of diabetic foot disease was estimated to cost the National Health Service (NHS) in England approximately £1 billion in 2014–2015, and this is increasing; annual submission of ulcer episodes by 221 specialist foot care services in England and Wales tripled from 2015 to 2018 (7965–15,370) (NHS Digital, 2019).

DFUs can be painful and cause loss of mobility, impacting on practical daily activities, increasing dependency on others and can lead to frustration and depression (Polikandrioti et al., 2020; Spanos et al., 2017). Less than half of all ulcers reported in the National Diabetes Foot Care Audit for England and Wales were healed at 12 weeks (48.7%), and people with severe ulcers were four times as likely to have a major amputation than less severe ulcers (SINBAD3 + 2.7%, SINB0-2 0.7%) (NHS Digital, 2019). Most wound care for DFUs is provided by nurses and other allied health professionals (AHPs) within the community and represents 62–64% of the cost of management of DFUs (Guest et al., 2018). The best outcomes are achieved with early diagnosis and effective treatment which involves services working closely with people with diabetes to prevent foot disease (NHS Digital, 2019).

The REDUCE programme was developed by a multidisciplinary team of clinical, academic and patient experts; patients develop skills to monitor changes in their feet, maintain safe levels of physical activity, seek early access to medical help and manage their mood (National Institute for Health Research (NIHR)).

As part of a programme of research into REDUCE (NIHR RP-DG-0615-10005 and RP-PG_0618-20001) (NIHR), value for money will be assessed. To develop a comprehensive economic evaluation requires accurate measurement of resource use, although this is often poorly reported, and instruments used are not commonly validated (Thorn et al., 2013). Often, measures are used from previous trials or studies, as they are or with modifications. There is increasing recognition that this is not good practice as it is important to choose measures and make modifications with careful consideration of the content and utility of this measure in a new population and trial setting. The importance of preparatory work to inform the resource-use measure (RUM) is increasingly recognised. Patient-reported measures should be tested for reliability, validity, responsiveness and interpretability (Ridyard and Hughes, 2012). A RUM should be sufficiently comprehensive, but still focused to capture the main drivers of resource use and associated cost to balance the trade-off between precision, effort and burden to the research participant and to help minimise missing data.

As part of the REDUCE research programme, we had the opportunity to develop a RUM specific to management of DFUs with input on the content and design from the research team, patient representatives and healthcare professionals (HCPs) who treat people with DFUs (Husereau et al., 2022). This measure was then tested in the pilot study for REDUCE to ensure validity for the patient population and disease area, and collection was viable. Here, we present the methodology for developing and validating the RUM.

## Objective

Our aim was to collaboratively explore key resource use related to the management of DFUs to develop, and pilot, a participant-reported RUM to inform economic evaluations.

## Study design

The process for developing a RUM has been described by Thorn et al. (2013). The first stage is a literature search to identify RUMs developed and the main cost elements in management of DFUs. The second stage involves discussion with HCPs and patients to determine the planned perspective, cost drivers and main resource items. From these discussions, a first draft RUM is developed which is tested for content and face validity by HCPs, patients and carers in a modified Delphi study. The RUM is revised after this stage and tested in the pilot study to assess acceptability, feasibility of collecting the economic outcomes and the completeness of data collection. The key drivers of resource use and costs are assessed, and appropriate face validity checks are applied to ensure accurate description of the treatment pathways. There are 10 core items that should be included in all RUMs including hospital care, emergency care, primary and community care, healthcare at home and medications, selected in a Delphi study for general healthcare resource-use questionnaires, which are included in the final RUM (Thorn et al., 2018).

A health economic analysis plan was developed prior to analysis of the pilot study conducted in SPSS (IBM v.15, U.S.A) and Excel (Microsoft 365, U.S.A).

## Methods

### Literature search

The Database of Instruments for Resource Use Measurement (DIRUM) and the NIHR library were searched to identify any relevant methods of collecting resource use in similar trials, or in a population with DFUs.

### Initial discussions

Researchers at the University of Southampton collated evidence from patients who had a DFU to plan the REDUCE intervention; the details of this study and the overall qualitative results have been reported previously (Greenwell et al., 2018). As part of the interview guide, three additional questions were asked to generate information on resources and costs associated with managing their DFUs. In addition to interviews with the patients, HCPs from five study centres (Nottingham, Derby, Ipswich, Bromley and Norwich) were asked to describe the most important cost drivers associated with DFUs. A broad perspective was taken to elicit information about cost to health services, social services, patients and their families and carers. A set of resource items was derived from these initial interviews to develop the RUM and reported as part of the final NIHR programme development grant report for RP-DG-0615-10005(summary available on request from authors).

### Modified Delphi study

To ensure the selected items represented a consensus view, we undertook a modified Delphi study (Hasson et al., 2000). The Delphi panel was formed of patients, carers and family members (up to *N* = 25) recruited from the REDUCE programme patient and public involvement group, other diabetes networks and with snowball sampling in November 2020. HCPs (up to *N* = 25) were recruited from current networks and contacts of the REDUCE researchers, and from HCPs who had registered an interest in the REDUCE programme, as well as being promoted via the REDUCE_DFU Twitter account. An invitation email was sent with a link to an online survey and consent forms.

Participants were asked to rate each item on a 9-point Likert scale according to the level of importance of the item for day-to-day management of DFUs, 1 indicated ‘not important’ and 9 indicated ‘very important’ (Akins et al., 2005; Vogel et al., 2019). Further space was provided alongside each item to enable comment (Stewart et al., 2017).

The second round required participants to rate the items where no consensus was reached in round one and rate any items that were added in the free-text responses. Given the resources available and further considerations regarding participants (e.g. participant fatigue), not more than three rounds were planned if consensus was not reached upon the remaining items (Keeney et al., 2006).

A formal sample size calculation was not required for this study. The Delphi method is a widely accepted research approach; however, there is no consensus upon the size of the panel required. A greater number of participants enhances the reliability of findings and reduces error (Keeney et al., 2011); however, Sekayi and Kennedy (2017) noted that numbers greater than 20–30 become unwieldly in an iterative process.

Descriptive statistical analysis was performed, including percentage of consensus upon each item, mean and standard deviation. Missing data on individual items were noted. Criteria for including items from round 1 into round 2 are reported in Table 1.

**Table 1.** Criteria for inclusion of items into the next round of modified Delphi study.

Criteria	Round 1	Round 2
A	Percentage of participants scoring 7–9, 50% or more	Percentage of participants scoring 7–9, 70% or more
B	Percentage of patients scoring 1–3, 15% or less	Percentage of patients scoring 1–3, 15% or less
C	Percentage of new items reported, 10% or more	Percentage of new items reported in round 2, 15% or more

To inform the consensus agreement of the Delphi method, we considered the level of agreement across participants and across rounds and documented the degree of stability in scores. To assess the inter-rater reliability between rounds, the intra-class correlation coefficient (ICC) was assessed for all items (two-way random effects model with ICC average measurement and absolute agreement between raters). This test is appropriate when we have multiple scores for a rater and wish to assess the agreement among raters (Koo and Li, 2016). An agreement of 0.75 or above was taken as an indication of good reliability, 0.5–0.75 as moderate and less than 0.5 as poor. A small change in scores was assumed to be a 1-point difference.

To compute the stability of the raters’ responses, Wilcoxon signed-rank tests were undertaken for the difference in responses between the two rounds. A *p*-value greater than 0.05 indicated no evidence that the results were different between the Delphi rounds.

### Pilot study

The responses from the modified Delphi study were used to revise the RUM for the pilot study. The multi-centre randomised controlled pilot study included adults, aged 18 years and older, with diabetes and a recently healed DFU and both lower limbs, recruited from specialist multidisciplinary diabetic foot clinics at participating secondary care NHS Trusts. The participant-reported RUM was included at baseline and the 4-month follow-up. The data collected at baseline was checked for face validity. Items with missing data were reported, with proportion of missing data, and patterns examined.

## Results

### Literature search

The comprehensive search identified 40 economic evaluations for full review. However, on full review, no suitable studies were identified that reported a full economic analysis relevant to the study question. Only one trial was identified which reported the use of a patient diary to collect health resource use, but several limitations were identified on the comprehensiveness of the measure (Jeffcoate et al., 2017).

### Focus groups

Semi-structured qualitative interviews were conducted with patients (*N* = 20) with a history of ulceration and 13 HCPs (nine podiatrists and four consultant diabetologists). From these groups, it was clear that an NHS perspective would not adequately address the resource use and costs that drive management of DFUs, with direct and indirect costs to the patient and family, including impact on employment, considered important. The HCPs highlighted the need for reporting different services and skill mix in delivering foot services across different sites. The responses from the interviews were used to develop a first draft of the RUM.

### Modified Delphi study

In total, 31 people expressed an interest and were invited to participate in the modified Delphi study, 25 HCPs and 6 people who had, or had previously had, a DFU. Most of the HCP group were podiatrists (*N* = 21), with two hospital doctors and two nurses also responding. Most HCP respondents were female (*N* = 21; 84%) and aged between 50 and 59 years (44%) (Table 2). The patient respondents were also mainly female (66.7%), and all respondents were aged 50 years and over.

**Table 2.** Demographics for the participants of the modified Delphi study (*N* = 6 patients, *N* = 25 healthcare professionals).

Characteristic	Patients	HCPs
Female	4 (66.7%)	21 (84.0%)
Age		
60–69	2 (33.3%)	1 (4.0%)
50–59	4 (66.7%)	11 (44.0%)
40–49	0	7 (28.0%)
30–39	0	4 (16.0%)
18–29	0	2 (8.0%)
Ethnicity		
White	5 (83.3%)	25 (100%)
Asian/Asian British	1 (16.7%)	0

HCPs: healthcare professionals.

In community care, podiatrist or orthotist consultations and district nurse visits were highly rated by patients and HCPs in both rounds. Community and primary care contacts were considered important to spot new symptoms, reduce isolation, and improve mental health and well-being. Generally, the ICC value for responses to each of the primary and community care items showed good to excellent inter-rater agreement (Table 3).

**Table 3.** Intra-class correlation coefficient, average measures and absolute agreement level (scores reported in Supplemental material) for resource-use items in round 1 and 2 of the modified Delphi study.

Item			
	Average measures (ICC) (95% confidence intervals)	Agreement level	Wilcoxon signed rank *p*-value
Community health care			
Face-to-face consultation with a podiatrist	0.848 (0.666, 0.929)	Good	0.248
Telephone/online consultation with a podiatrist	0.699 (0.333, 0.863)	Good	0.761
Home visit by a podiatrist	0.829 (0.622, 0.922)	Good	0.584
Home visit by a district nurse	0.838 (0.649, 0.926)	Good	0.288
Home visit by a social care/personal care (home help) assistant	0.561 (0.024, 0.801)	Moderate	0.777
Face-to-face consultation with an orthotist at a community centre/workshop or rehabilitation centre	0.366 (−0.273, 0.698)	Poor	0.035
Face-to-face consultation with other community healthcare professional (see Table 4)			
Telephone/online consultation with other community healthcare professional	0.753 (0.468, 0.886)	Good	0.142
Out-of-hours/emergency care			
Telephone or online contact with NHS 111/NHS 24	0.701 (0.336, 0.865)	Moderate	0.877
Telephone or online contact with GP service	0.749 (0.442, 0.886)	Moderate	0.902
Face-to-face consultation with GP service at community centre or at emergency department/hospital setting	0.675 (0.303, 0.850)	Moderate	0.246
Walk-in/Minor injury unit	0.665 (0.256, 0.848)	Moderate	0.680
Attendance at home and transferred to hospital (emergency/A&E; department)	0.583 (0.067, 0.812)	Moderate	0.979
Hospital care			
Face-to-face attendance with orthotic clinic	0.227 (−0.634, 0.642)	Poor	0.105
Attendance for scan/investigations (see Table 4)			
Inpatient stay at other NHS/Social Care unit for example, rehabilitation unit, intermediate care unit	0.622 (0.187, 0.826)	Moderate	0.275
Prescriptions/Equipment			
Prescribed/provided in clinic dressings/wound healing preparations for DFU	0.718 (0.394, 0.870)	Moderate	0.236
Prescribed/provided in clinic devices for DFU (e.g. insoles, off-loading devices)	0.428 (−0.248, 0.739)	Poor	0.284
Equipment provided for example, walking stick, crutches, wheelchair, home adaptations such as raised toilet seat, pressure relieving cushion (see Table 4)			
Personal costs			
Travel to appointments (for example, public transport, taxi) by yourself/family/friend	0.361 (−0.434, 0.712)	Poor	0.337
NHS ambulance car/patient transport service (not ‘999’)	0.731 (0.403, 0.878)	Moderate	0.886
Time off work to attend appointments, paid and unpaid	0.203 (−0.807, 0.642)	Poor	0.286
Time off work/sick leave related to DFUs, paid and unpaid	0.441 (−0.250, 0.747)	Poor	0.214
Time for informal carer when patient is ill due to DFUs	0.676 (0.296, 0.852)	Moderate	0.320
Time for informal carer to accompany patient to appointments	0.804 (0.568, 0.911)	Good	0.804
Time for informal carer for patient's dependent when you are admitted to hospital	0.726 (0.411, 0.874)	Moderate	0.289
Disability (personal) living allowance if patient not in paid work	0.857 (0.686, 0.935)	Good	0.572
Carer in receipt of allowance	0.643 (0.212, 0.838)	Moderate	0.458
Early retirement due to DFUs	0.828 (0.624, 0.921)	Good	0.441

Out-of-hours and emergency care, visits to walk-in or minor injury units, and attendances by a paramedic/ambulance and transferred to hospital were considered important by all respondents. The ICC for these responses also showed good to excellent agreement from both HCPs and patients (Table 3). Scans and investigations were also consistently highly rated, with computed tomography (CT) scans and non-invasive tests, for example toe pressure, rated highest by the patients; X-rays, magnetic resonance imaging and duplex arterial scans, were rated highest by HCPs (Table 4).

**Table 4.** New resource-use items added in free text of modified Delphi study (scores reported in Supplemental material).

Item
Face-to-face consultation with psychologist
Face-to-face consultation with counsellor
X-ray
MRI
Duplex arterial scan
CT scan
Ultrasound scan
MRA scan
CT angiogram
Non-invasive test
Pressure relieving equipment provided
Mobility equipment provided
Off-loading equipment provided

CT: computed tomography; MRI: magnetic resonance imaging.

Consumables were also highly rated, with dressings, wound healing preparations, devices for DFUs provided in clinic, and mobility equipment, considered most important for management. The ICC for prescribed/provided in clinic dressings/wound healing preparations was good (0.718), however when considering devices provided in clinic for DFU (for example, insoles, off-loading devices) the agreement became insignificant.

Informal care provided when a patient is ill due to DFUs, to accompany a patient to an appointment, or to provide care for a dependent when the patient is admitted to hospital, were considered important for inclusion by the HCPs but not the patients. The same was found for items related to disability living allowance, a carer in receipt of allowance, or early retirement due to DFUs.

Items that were poorly rated were excluded from round 2 (Table 5). Physiotherapist or occupational therapist visits in the community had low scores in round 1, although it was also noted that some services are linked, for instance physiotherapy or occupational therapy clinics are offered alongside other services such as smoking cessation, weight management, mental health services and social care services.

**Table 5.** Resource-use items excluded after low scores in modified Delphi study (scores reported in Supplemental material).

Item
Telephone/online consultation with a district nurse
Face-to-face consultation with a social worker at a community centre/local authority venue
Telephone/online consultation with a social worker
Home visit by a social worker
Face-to-face consultation with a physiotherapist or occupational therapist at a community centre
Telephone/online consultation with a physiotherapist/occupational therapist
Home visit by a physiotherapist/occupational therapist
Attendance at a local authority/NHS day care centre
Attendance at home by paramedic/ambulance but NOT transferred to hospital
Own purchase of pain relief, creams, etc.
Own purchase of dressings
Own purchase of equipment
Private chiropodist
Private orthotic service
Private complementary treatment
Other private healthcare
Own paid for cost of residential/nursing home accommodation
Own paid for care at home

NHS: national health service.

### Pilot study validation

The results of the modified Delphi study were used to revise the RUM which was delivered in the REDUCE pilot study involving 20 participants. Most participants were white (95%), male (70%), retired (65%) and married (65%).

Community care or outpatient appointments in the last 3 months were completed, although there was missing data for the number of appointments (*N* = 10 missing data points, 1.25%) (Table 6). Bandages, dressings, and gauze were the most reported in the medicines and dressings section (23 out of 27 items reported), and these were most likely to be prescribed or provided in clinic. Insoles, boots, and shoes were the most reported devices (7 out of 9 devices reported). The personal costs section had the most non-responses, with between one and three people not responding to individual questions in this section (Table 6).

**Table 6.** Resource items included in the resource use measure and missing data in the REDUCE pilot study, *N* = 20 participants.

Resource item	Missing data points *N*
GP and practice nurse appointments – face to face/online/phone	4 number of appointments not reported
Podiatrist – community or home	3 number of appointments and length not reported
Other community services – district nurse, orthotist, other services – face to face/online/phone	1 number of appointments not reported
Outpatient appointments and day cases – orthotics, physiotherapist, occupation therapist, other foot health	4 number of appointments not reported
Emergency care – A&E, paramedic/ambulance	1 person did not respond to any questions
Inpatient admissions	1 person did not respond to any questions
Scans and imaging	1 person did not respond to any questions
Out-of-hours – NHS111/NHS24, GP, walk-in or minor injury unit	1 person did not respond to any questions, 1 person did not respond to questions on the walk-in/minor injury units
Prescriptions – free, paid for and non-prescription	1 person did not respond to any questions, 2 additional people did not respond to the question on free prescriptions
Medicines/dressings/wound preparation	7 people did not report
Devices	N/A
Equipment – pressure relieving, mobility, off-loading	3 people did not report pressure relieving equipment, 6 people did not report mobility equipment, 6 people did not report off-loading equipment
Personal costs – early retirement, time off work, time for family and friends, transport	2 people did not respond to the question on time off for appointments, 1 person did not report sick leave or carers stopping work, 3 people did not report on friend/family member support

The location of appointments was not always clear, for instance orthotic clinics appeared to be reported twice, in the community and hospital sections. Given these responses the questionnaire was re-worded to ask about each healthcare professional/service, with the location asked as part of the question. There were several items where no visits or attendances were recorded in the pilot study: General practitioner (GP) home visits, ambulance attendances, out-of-hours contacts and inpatient stays.

## Discussion

DFUs place a considerable burden on patients, their families and carers, and healthcare services. Understanding the full resource use and costs related to DFUs will enable comprehensive economic evaluation. The REDUCE research programme provided the opportunity to develop a condition-specific RUM for DFUs that is extensive enough to capture all relevant cost drivers and is viable for inclusion in research, in terms of acceptability. Engaging people with experience of DFUs and HCPs to understand the impact from their viewpoints is an important first step in the design of any participant-reported outcome measure including economic outcome measures.

Primary or community care consultations with a podiatrist or orthotist were highly rated by patients and HCPs, as were out-of-hours and emergency care services. In hospital, scans and investigations were highly rated by patients and HCPs. Consumables were also highly rated by both groups, unsurprisingly dressing and wound healing preparations provided in clinics the highest rated. Interestingly, informal care if a patient is ill due to DFUs or admitted to hospital, or to accompany patients to hospital, disability living allowance and early retirement were not considered important by the patients but were by the HCPs.

**Figure 1.**
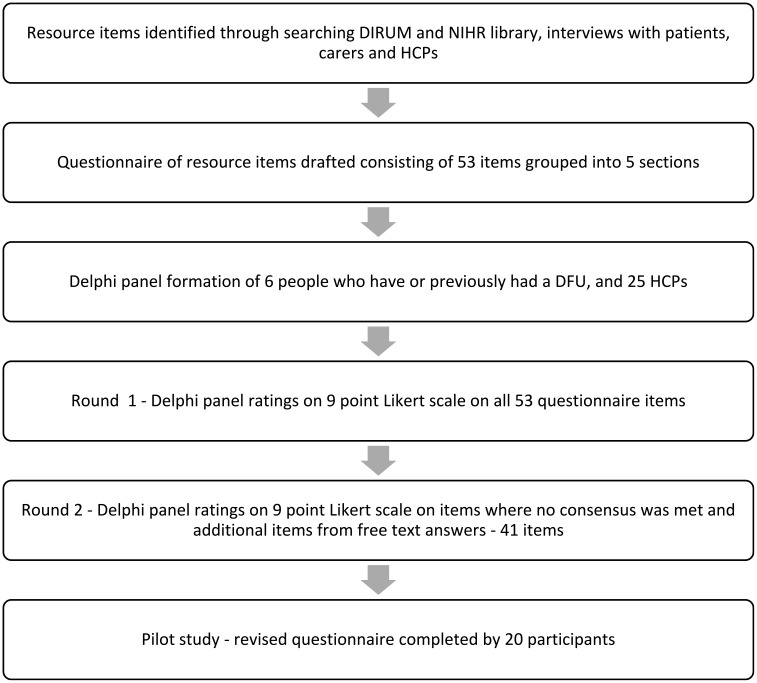
Flow chart of the resource use measure development process.

The RUM developed through the interviews and modified Delphi method was then tested in the pilot study for REDUCE to determine if the RUM was written in a clear and understandable way to allow accurate responses (Figure 1). Response rates were good for healthcare resources with limited missing data, personal costs were not as well-reported, which may be due to non-response if these items were not relevant to the participant (Table 6). There were some items with no recorded contacts which were discussed with the research team and due to the short time horizon of the pilot study, and that these items were identified in the interviews with patients and HCPs, it was decided that they should remain in the final RUM. Some items were reported twice due to confusion over location of services, and these questions were re-worded to include the location.

A limitation of the RUM development was the small number of patient participants in the modified Delphi study, with overall responses likely to be skewed by the HCP participants. However, 20 patients with a history of ulceration were interviewed to develop the initial matrix, and 20 participants were included in the pilot study, which may redress the balance.

A comment was received by an HCP regarding the potential for virtual appointments for some services; however, online and telephone appointments were not highly rated in the questionnaire. The modified Delphi study was carried out in November 2020, and England was in a second lockdown at this time; there would have been considerable disruption to healthcare services, which continues to this day. Virtual appointments may become more common and would need to be factored into the RUM in the future.

A checklist for reporting of economic evaluations (CHEERS) (Husereau et al., 2022), includes the ‘approach to engagement with patients and others affected by the study’. Developing a RUM with patients, family members and carers, and HCPs promotes engagement in research; these groups are the experts on their health and provide valuable insight into which economic outcomes should be considered for research within standard frameworks. An evaluation of lightweight fibre-glass heel casts in the management of ulcers in diabetes used a patient log to capture resource use; however, the data were found difficult to interpret due to the broad categories included (Jeffcoate et al., 2017). The preparatory work in developing a validated RUM for management of DFUs will enable us to better describe resource use in detail to inform future economic evaluations relevant to decision-makers within the NHS and wider society.

## Conclusion

A comprehensive economic evaluation requires accurate measurement of resource use and associated costs. Compared to the research efforts on appropriate measurement of outcomes for economic evaluation, research into measuring resource use has been neglected (Thorn et al., 2013).

Patients, their family and carers, and HCPs, with nurses and other AHPs in the community providing the majority of wound care for this group, are the experts in the resource impact of DFUs. This research, as part of the REDUCE programme, has enabled development of a comprehensive, valid and viable RUM for management of DFUs to inform development of economic evaluation alongside the main trial. Input from key stakeholders ensures accurate and relevant resource use measurement for economic evaluations that will provide valuable evidence for decision-making within the health and social care sectors. We believe this is the first RUM specifically for DFUs which has been designed with formal involvement and engagement of a range of stakeholders, and it will be made freely available on the DIRUM website for other researchers.

**Key points for policy, practice and/or research**

- Patients, family members and carers are the experts on the impacts of their health states and should be involved in development of patient reported resource-use measures (RUMs).
- HCPs, with nurses and other AHPs providing the majority of wound care for this group of patients, can provide valuable insight into development of RUMs to inform economic evaluation.
- RUMs should be comprehensive to allow accurate costing but should not be a burden to respondents.
- Preparatory work to develop a RUM specific to the disease area and population informs better quality and more relevant economic evaluations.

## Supplemental Material

sj-pdf-1-jrn-10.1177_17449871231208108 – Supplemental material for Development of a resource-use measure to capture costs of diabetic foot ulcers to the United Kingdom National Health Service, patients and society Click here for additional data file. Supplemental material, sj-pdf-1-jrn-10.1177_17449871231208108 for Development of a resource-use measure to capture costs of diabetic foot ulcers to the United Kingdom National Health Service, patients and society by Katherine Cullen, Mari Jones, Christina Sheehan, Frances Game, Kavita Vedhara and Deborah Fitzsimmons in Journal of Research in Nursing

sj-pdf-2-jrn-10.1177_17449871231208108 – Supplemental material for Development of a resource-use measure to capture costs of diabetic foot ulcers to the United Kingdom National Health Service, patients and society Click here for additional data file. Supplemental material, sj-pdf-2-jrn-10.1177_17449871231208108 for Development of a resource-use measure to capture costs of diabetic foot ulcers to the United Kingdom National Health Service, patients and society by Katherine Cullen, Mari Jones, Christina Sheehan, Frances Game, Kavita Vedhara and Deborah Fitzsimmons in Journal of Research in Nursing

sj-pdf-3-jrn-10.1177_17449871231208108 – Supplemental material for Development of a resource-use measure to capture costs of diabetic foot ulcers to the United Kingdom National Health Service, patients and society Click here for additional data file. Supplemental material, sj-pdf-3-jrn-10.1177_17449871231208108 for Development of a resource-use measure to capture costs of diabetic foot ulcers to the United Kingdom National Health Service, patients and society by Katherine Cullen, Mari Jones, Christina Sheehan, Frances Game, Kavita Vedhara and Deborah Fitzsimmons in Journal of Research in Nursing
