# Supplementary material for: Development of a resource-use measure to capture costs of diabetic foot ulcers to the United Kingdom National Health Service, patients and society
Source: J Res Nurs. 2023 Dec 27;28(8):565–78. doi: 10.1177/17449871231208108 (PMC10756167; doi:10.1177/17449871231208108)
Supplement: sj-pdf-2-jrn-10.1177_17449871231208108 – Supplemental material for Development of a resource-use measure to capture costs of diabetic foot ulcers to the United Kingdom National Health Service, patients and society [file sj-pdf-2-jrn-10.1177_17449871231208108.pdf]

HCP, Person with DFU, Carer & Family Member Participant Information Sheet (Final v2.0, 8 October 2020)

Title of Study: Development of the set of resource items for a questionnaire to capture costs of diabetic foot ulcers (DFUs) to the NHS, patient and society for the REDUCE trial: Delphi Study

IRAS Project ID: 286316

We invite you to take part in a research study to help us develop a measure to capture resource use in diabetic foot ulcer (DFU) care for the NHS, patients and society.

- Before you decide whether to take part, it is important for you to understand why the research is being done and what it will involve.
- Please take time to read the following information carefully. Discuss it with friends and relatives if you wish.
- You are free to decide whether or not to take part in this research. If you choose not to take part, this will not affect your legal rights.
- Ask us if there is anything that is not clear or if you would like more information.
- In this research study we will use information from you. We will only use information that we need for the research study. We will let very few people know your name or contact details, and only if they really need it for this study.
- Everyone involved in this study will keep your data safe and secure. We will also follow all privacy rules.
- At the end of the study we will save some of the data in case we need to check it and for future research.
- We will make sure no-one can work out who you are from the reports we write.
- This information sheet tells you more about this.

Why are we doing this study?

Researchers have developed an intervention called REDUCE which targets the psychological and behavioural risk factors associated with diabetic foot ulcer (DFU) recurrence and healing. Determining the cost of REDUCE and whether this cost is justified when compared against usual care is required as part of a future trial. The purpose of this study is ensure the set of questions to be included in the questionnaire represents a consensus view from people who have had DFUs or the people living and/or caring for them . This will help us identify which items are most important to capture the resources and associated costs of DFUs from the perspective of the NHS, the patient and society.

Our study aims:

(a) To agree a set of items for inclusion in a participant-reported measure to capture resource use for the NHS, patient and society in DFU care.

(b) To construct a participant reported measure to capture resource use and costs for piloting as part of the REDUCE pilot trial.

Why am I being asked to take part?

We would like up to 25 health professionals involved in, or previously involved in diabetic foot ulcer care, and up to 25 people with DFUs, their carers and/or family members to take part in this study.

What will I do if I take part?

- We will ask you to complete 2-3 online questionnaires over the next 1-2 months.
- Each questionnaire will take approximately 30 minutes. A total of 60-90 minutes for the 2-3 questionnaires.
- You can rate each item in the questionnaire depending upon how important you think it is on a scale of 1-9 (1 = not important, 9 = very important). There will be space next to each item for you to write any comments or thoughts on the items.
- In addition we will ask for some background information including your sex, age group and ethnic group. If you are a healthcare professional we will also ask for your job category, e.g. podiatrist, nurse, orthotist, hospital doctor, GP. We use this data to ensure the groups are representative.
- We will also require your contact details (name and email address) to send future questionnaires.
- You will receive an electronic link to the second (and if required, the third) questionnaire via email, approximately 2 weeks after the previous questionnaire.
- We will send you up to two email reminders to complete the second (and if required, the third) questionnaire.

What are the benefits and risks of taking part?

Your participation will help develop the resource use questionnaire for the future REDUCE trial and will ensure the items used are relevant to NHS professionals, people with DFUs, and carers and family members of people with DFUs.

There are no significant risks anticipated by taking part in this study. Your involvement will be for approximately 90 minutes in total (around 30 minutes per questionnaire).

How will we use information about you?

We will need to use information from you for this research project.
This information will include your:

- Name
- Email address
- Sex
- Ethnic Group
- Age Group

People will use this information to do the research or to check your records to make sure that the research is being done properly.

People who do not need to know who you are will not be able to see your name or contact details. Your data will have a code number instead.

We will keep all information about you safe and secure.

Once we have finished the study, we will keep some of the data so we can check the results. We will write our reports in a way that no-one can work out that you took part in the study.

Members of the research team at University Hospitals Derby and Burton NHS Foundation Trust, the University of Nottingham and regulatory authorities will see the information in this list and the questionnaire data. The information will be password-protected. The University of Nottingham will store your personal details on physical servers based on the campus, backed up every 24 hours. Your questionnaire responses without your personal details will be stored on Microsoft OneDrive/Teams cloud storage on servers based in the UK. This data will have a personal study code to identify you. Researchers on this study at Swansea Centre for Health Economics at Swansea University (who are conducting the analysis) and University Hospitals of Derby and Burton NHS Foundation Trust (the sponsor) will also be able to access the questionnaire data, but will not be able to see any personal details as these will stored separately, on a different non-cloud based server. For more information on how Microsoft store data, please see here: https://docs.microsoft.com/en-us/office365/enterprise/o365-data-locations#data-center-locations.

Online Surveys will also store the information listed and the questionnaire data in their data centres in the Republic of Ireland. Backups of data are stored in the United Kingdom and are held for a maximum of three months. After three months the backups are deleted and destroyed. For more information about how Online Surveys stores data, please see here: https://www.onlinesurveys.ac.uk/help-support/online-surveys-security/#:~:text=Online%20surveys%20has%20a%20data,backups%20are%20deleted%20and%20destroyed.

We will keep the data for 5 years after the end of the study.

The sponsor of the research University Hospitals of Derby and Burton NHS Foundation Trust is also responsible for ensuring your personal information is kept confidential. If you require further information as to how your information is handled as part of research then you can find it online here: https://www.uhdb.nhs.uk/research-how-we-use-your-information or contact the Data Protection Officer Anne Woodhouse via email at: Anne.Woodhouse1@nhs.net

What are your choices about how your information is used?

- You can stop being part of the study at any time, without giving a reason, but we will keep information about you that we already have.
- We need to manage your records in specific ways for the research to be reliable. This means that we won't be able to let you see or change the data we hold about you.

- If you change your mind and do not wish to receive future questionnaires, you can contact the research team using the contact details below.

Where can you find out more about how your information is used?

You can find out more about how we use your information:

- by asking one of the research team (contact details below)
- by sending an email to christina.sheehan@nottingham.ac.uk
- by ringing us on 0115 8230455
- at https://www.hra.nhs.uk/information-about-patients/
- at www.hra.nhs.uk/planning-and-improving-research/policies-standards-legislation/data-protection-and-information-governance/gdpr-guidance/templates/template-wording-for-generic-information-document/

What will happen to the results of this study?

The results from this study will be used to develop the resource use questionnaire for the REDUCE trial. The findings will be included in reports to the funder of the Programme, and will be presented to the REDUCE programme management group, independent steering committee, and patient and public involvement group. We may publish the results in academic journals and present the findings at academic conferences. We will write our reports, presentations and publications in a way that no-one can work out that you took part in the study.

Who is organising and funding this study?

This study is sponsored by University Hospitals Derby and Burton NHS Foundation Trust (UHDB) and is a collaboration between researchers at UHDB, the University of Nottingham and Swansea Centre for Health Economics at Swansea University.

This study is funded by the National Institute for Health Research (NIHR) and is part of the REDUCE Programme Grant for Applied Research.

How have patients and the public been involved in this study?

The REDUCE programme has a dedicated patient and public involvement group who meet three times per year and have reviewed and provided input into the study documents.

Who has reviewed this study?

This study has been reviewed by the Health Research Authority and Yorkshire & The Humber - Bradford Leeds Research Ethics Committee (IRAS ref: 286316; Ethics ref: 20/YH/0286).

Who can I contact if I have any questions or concerns about the study?

If you have any other questions about this research, please contact the research team using the details below:

REDUCE Programme Manager: Christina Sheehan
Phone: 0115 8230455
Email: christina.sheehan@nottingham.ac.uk

If you wish to make a complaint, please contact the Chief Investigator via the email address below:

Chief Investigator: Professor Fran Game
Email: reduce@nottingham.ac.uk

Thank you very much for your time reading this information. If you wish to take part please [enter website instructions/proceed to next page]
