# Supplementary material for: Development of a resource-use measure to capture costs of diabetic foot ulcers to the United Kingdom National Health Service, patients and society
Source: J Res Nurs. 2023 Dec 27;28(8):565–78. doi: 10.1177/17449871231208108 (PMC10756167; doi:10.1177/17449871231208108)
Supplement: sj-pdf-3-jrn-10.1177_17449871231208108 – Supplemental material for Development of a resource-use measure to capture costs of diabetic foot ulcers to the United Kingdom National Health Service, patients and society [file sj-pdf-3-jrn-10.1177_17449871231208108.pdf]

Development of the set of resource items for a questionnaire to capture costs of diabetic foot ulcers (DFUs) to the NHS, patient and society for the REDUCE trial: Delphi Study	
**Short title: REDUCE: Delphi study to develop resource use questionnaire (RUQ)**	
**Version and Date of Protocol:**	Final v1.0, 2 September 2020
**Sponsor:**	University Hospitals of Derby & Burton NHS Foundation Trust
**Chief Investigator:**	Professor Fran Game
**Local Reference:**	UHDB/2020/087
**IRAS Number:**	286316
**Funder(s):**	National Institute for Health Research (NIHR) Programme Grant for Applied Research (PGfAR) (Reference: RP-PG-0618-20001). This protocol is part of the Health Economics Work Package of the REDUCE Programme Grant.
**This protocol has regard for the HRA guidance**	

Confidentiality Statement

This document contains confidential information that must not be disclosed to anyone other than the Sponsor, the Investigator Team, host NHS Trust, regulatory authorities, and members of the Research Ethics Committee.

SIGNATURE PAGE

The undersigned confirm that the following protocol has been agreed and accepted and that the Chief Investigator agrees to conduct the study in compliance with the approved protocol and will adhere to the principles outlined in the Declaration of Helsinki, the Sponsor's SOPs, and other regulatory requirement.
I agree to ensure that the confidential information contained in this document will not be used for any other purpose other than the evaluation or conduct of the investigation without the prior written consent of the Sponsor.
I also confirm that I will make the findings of the study publically available through publication or other dissemination tools without any unnecessary delay and that an honest accurate and transparent account of the study will be given; and that any discrepancies from the study as planned in this protocol will be explained.

Protocol, final v1.0, 2 September 2020, authorisation signatures:

Chief Investigator:

Signature:	………………………………………………………..	Date: ………/………/………………
Name (please print):	………………………………………………………..	

For and on behalf of the Study Sponsor (if required):

Signature:	………………………………………………………..	Date: ………/………/………………
Name (please print):	………………………………………………………..	
Position:	………………………………………………………..	

KEY STUDY CONTACTS

Chief Investigator:	Professor Fran Game, Royal Derby Hospital<br>University Hospitals of Derby and Burton NHS Foundation Trust.<br>Tel: +44 (0)1332 724736<br>Email: fran.game@nhs.net
Co-Investigator(s):	Professor Deborah Fitzsimmons, Swansea Centre for Health Economics, College of Human and Health Sciences, Office 214, Second Floor, Vivian Building, Swansea University, Singleton Campus, Swansea, SA2 8PP<br>Tel: +44 (0)1792 602226/07803 044029<br>Email: d.fitzsimmons@swansea.ac.uk<br><br>Ms Katherine Cullen, Swansea Centre for Health Economics, College of Human and Health Sciences, Room: SCHE – 218, Second Floor, Vivian Building, Singleton Campus, Swansea, SA2 8PP<br>Email: katherine.cullen@swansea.ac.uk<br><br>Mrs Christina Sheehan, Division of Primary Care, University of Nottingham, University Park, Nottingham, NG7 2RD<br>Tel: +44 (0)115 8230455<br>Email: christina.sheehan@nottingham.ac.uk<br><br>Ms Rebecca Summers, Swansea Centre for Health Economics, College of Human and Health Sciences, Room: SCHE – 218, Second Floor, Vivian Building, Singleton Campus, Swansea, SA2 8PP<br><br>Professor Kavita Vedhara, Division of Primary Care, University of Nottingham, University Park, Nottingham, NG7 2RD<br>Tel: +44 (0)115 8466931<br>Email: kavita.vedhara@nottingham.ac.uk
Study Co-ordinator:	Mrs Christina Sheehan, Division of Primary Care, University of Nottingham, University Park, Nottingham, NG7 2RD<br>Tel: +44 (0)115 8230455<br>Email: reduce@nottingham.ac.uk
Sponsor:	University Hospitals of Derby & Burton NHS Foundation Trust<br>Royal Derby Hospital<br>Uttoxeter Road<br>Derby, DE22 3NE<br>01332 724639<br>UHDB.sponsor@nhs.net

Funder(s):	The study is funded by the National Institute for Health Research (NIHR) Programme Grants for Applied Research (PGfAR), reference RP-PG-0618-20001.
Study Statistician:	Ms Katherine Cullen, College of Human and Health Sciences, Room: SCHE – 218, Second Floor, Vivian Building, Singleton Campus, Swansea, SA2 8PP<br>Tel: +44 (0) 1792 295789<br>Email: katherine.cullen@swansea.ac.uk

STUDY SUMMARY

**Study Title:**	Reducing the impact of diabetic foot ulcers (DFUs): Development of the set of resource items for a questionnaire to capture costs of DFUs to the NHS, patient and society for the REDUCE trial: Delphi Study
**Short title:**	Reducing the impact of DFUs: Delphi Study
**Local Study Reference:**	UHDB/2020/087; IRAS 286316
**Study Design:**	Delphi Study
**Study Participants:**	Healthcare Professionals (HCPs) working in the area of DFU care and patient and public involvement representatives (people with DFUs, their carers and/or family members)
**Planner Number of Sites:**	N/A
**Planned Sample Size:**	N=50 Up to 25 patient representatives (e.g. people with DFUs, carers and/or family members of people with DFUs), and up to 25 HCPs working in the area of DFU care.
**Follow Up Duration:**	2 months
**Planned Start Date:**	28/09/2020
**Planned Recruitment End Date:**	30/10/2020
**Planned Study End Date:**	31/12/2020
**Research Question/ Aims:**	To agree a set of items for inclusion in a participant-reported measure to capture resource use for NHS, patient and society in DFU care.

FUNDING AND SUPPORT IN KIND

Funder(s)	Financial and Non-Financial Support Given
NIHR PGfAR (Reference: RP-PG-0618-20001)	£2,531,202
Swansea Centre for Health Economics – Swansea University	Non-financial – supported as part of commitment to the REDUCE Programme

ROLES & RESPONSIBILITIES

Sponsor
The Sponsor, University Hospitals of Derby & Burton NHS Foundation Trust, take on overall responsibility for appropriate arrangements being in place to set up, run and report the research project. The sponsor is not providing funds for this study, but has taken on responsibility for ensuring finances are in place to support the research.

Funder
The study is funded by the National Institute for Health Research (NIHR) Programme Grants for Applied Research (PGfAR), reference RP-PG-0618-20001.

STUDY MANAGEMENT COMMITTEES

Programme Management Group (PMG)
The study is part of a larger programme of work which will be overseen by the REDUCE PMG. The PMG consists of all the applicants and collaborators on the wider programme and one lay/PPI member. The PMG will meet bi-annually over the programme to oversee the management of all work packages (WPs), including the work described here. The PMG will be notified of any problems with study conduct.

Independent Programme Steering Committee (IPSC)
The IPSC oversee and supervise the progress of the programme and convene every 6-12 months. The IPSC consists of five members; an independent Chair with expertise in health psychology, an independent clinician with expertise in diabetes, an independent member with expertise in podiatry, an independent statistician and an independent patient and public involvement (PPI) representative. The Programme Co-Chief Investigators (the Chief Investigator and Professor of Health Psychology on this study) and Programme Manager will attend the IPSC to provide the updates. A Sponsor representative and a Funder representative may also be in attendance. Other members will be invited on an ‘as required’ basis.

Protocol Contributors
A number of protocol contributors have been involved in the development of this protocol. These include; the Chief Investigator (Consultant Diabetologist), Lead Health Economist (Professor of Health Outcomes Research), Health Economist, Lead Health Psychologist (Professor of Health Psychology) & Programme Manager.

Contents

SIGNATURE PAGE 2
KEY STUDY CONTACTS 3
STUDY SUMMARY 5
FUNDING AND SUPPORT IN KIND 6
ROLES & RESPONSIBILITIES 6
LIST OF ABBREVIATIONS 9
STUDY FLOW CHART 10
1. BACKGROUND 11
2. RATIONALE 13
3. OBJECTIVES AND OUTCOME MEASURES/ ENDPOINTS 14
3.1. Objectives 14
4. STUDY DESIGN 14
5. STUDY SETTING 14
6. ELIGIBILITY CRITERIA 15
6.1. Inclusion Criteria 15
6.2. Exclusion Criteria 15
7. STUDY PROCEDURES 15
7.1. Recruitment 15
7.1.1. Participant Identification 16
7.2. Consent 16
7.3. Study Assessments 16
7.3.1. First Round 17
7.3.2. Second and Third Rounds 17
7.4. Withdrawal Criteria 17
7.5. End of Study 17
8. SAFETY REPORTING 17
9. DATA HANDLING 18
9.1. System and Compliance 18
9.2. Source Data 18
9.3. Data Workflow, Access and Security 18
9.4. Archiving 18
10. STATISTICS AND DATA ANALYSIS 19
10.1. Sample Size Calculation 19
10.2. Statistical Analysis 19
10.3. Procedure(s) to Account for Missing or Spurious Data 19
11. MONITORING, AUDIT & INSPECTION 19
12. ETHICAL AND REGULATORY CONSIDERATIONS 19
12.1. Assessment and Management of Risk 19
12.2. Peer review 19
12.3. Public and Patient Involvement 20
12.4. Research Ethics Committee (REC) & Regulatory Considerations 20
12.5. Protocol Compliance / Non-compliance Reporting 20
12.6. Notification of Serious Breaches to GCP and/or the Protocol 20
12.7. Data Protection and Participant Confidentiality 21
12.8. Financial and Other Competing Interests for the Chief Investigator, Principal Investigators at Each Site and Committee Members for the Overall Study Management 21
12.9. Indemnity 21
12.10. Amendments 21
12.11. Access to Final Study Dataset 22

13. DISSEMINATION POLICY 22
13.1. Dissemination Policy 22
13.2. Authorship Eligibility Guidelines and any Intended Use of Professional Writers 22
14. REFERENCES 22

LIST OF ABBREVIATIONS

AE	Adverse Event
CI	Chief Investigator
DFU	Diabetic Foot Ulcer
DMEC	Data Monitoring and Ethics Committee
GCP	Good Clinical Practice
GDPR	General Data Protection Regulator
HCP	Healthcare Professional
IP	Internet Protocol
IPSC	Independent Programme Steering Committee
NHS	National Health Service
NHS R&D	National Health Service Research & Development
NIHR	National Institute for Health Research
PGfAR	Programme Grant for Applied Research
PICO	Patient/Problem/Population, Intervention, Comparison/Control/Comparator, Outcome(s)
PIS	Participant Information Sheet
PMG	Programme Management Group
PPI	Patient and Public Involvement
PSS	Personal Social Services
REC	Research Ethics Committee
SCHE	Swansea Centre for Health Economics
SOP	Standard Operating Procedure
SMF	Study Master File
UoN	University of Nottingham

STUDY FLOW CHART

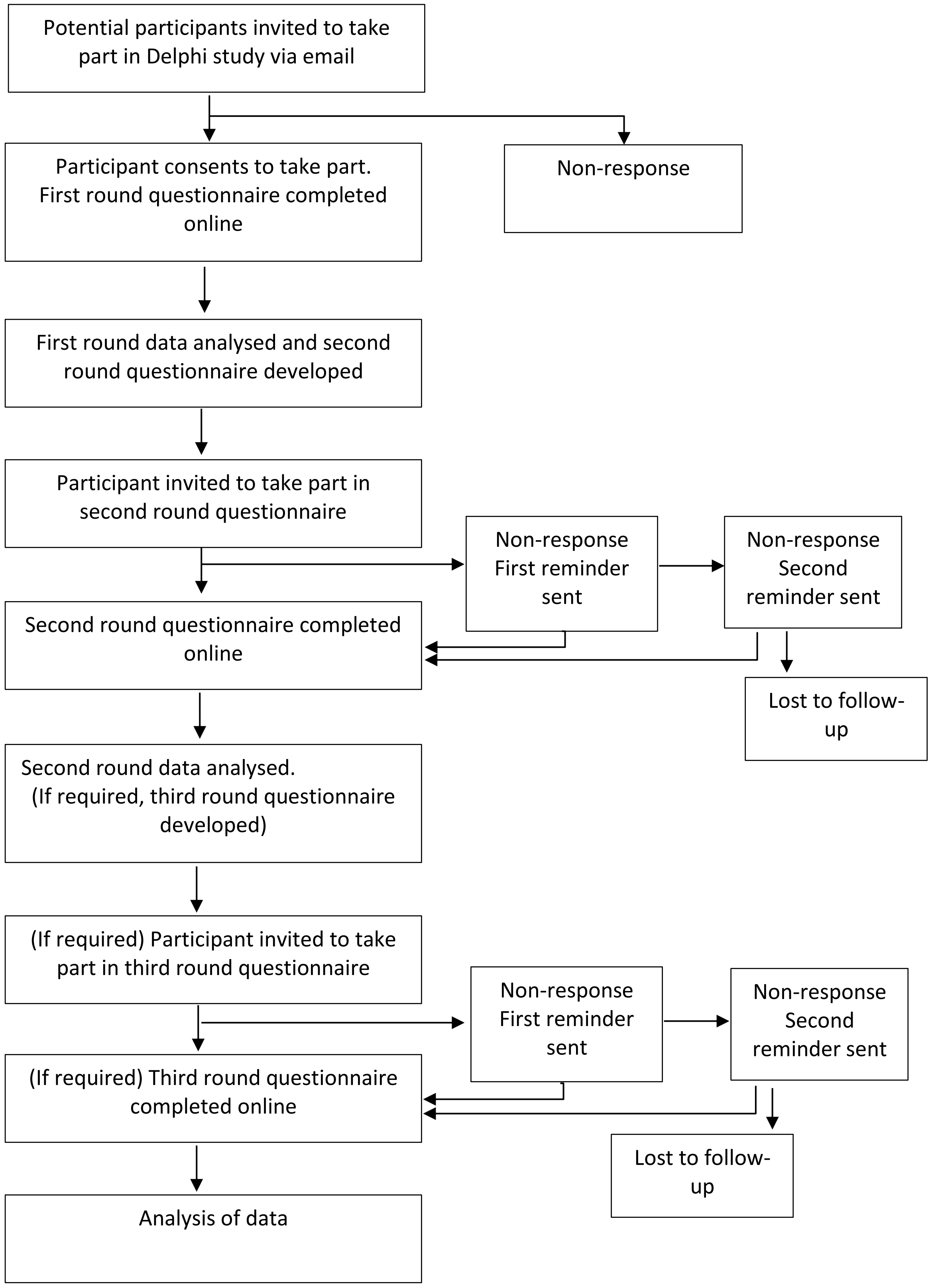

STUDY PROTOCOL

1. BACKGROUND

REDUCE is a complex intervention which targets the psychological/behavioural risk factors associated with diabetic foot ulcer (DFU) recurrence and healing. As part of a comprehensive evaluation, determining the cost-effectiveness of REDUCE is required and the REDUCE trial will include a health economic evaluation alongside the main trial. As part of the economic analysis, we require the collection of participant level resource use associated with the REDUCE intervention compared with usual care. Our main perspective will be UK NHS/Personal Social Services (PSS) but we also will collect information to undertake an additional analysis of the costs to the patient and (from a limited perspective) society.

There is a variety of data collection methods for capturing data on resource use. These include patient self-report (e.g. questionnaires, diaries or interviews), use of routine data (e.g. medical records) and use of expert panels. All methods come with advantages and disadvantages. Whilst the REDUCE trial will consider whether resource use data can be obtained from sources such as trial records in order to reduce burden and bias, a resource use measure is required to standardise data collection.

Compared to other patient self-reported outcomes, methods for collecting resource use vary widely. Often, measures are used from previous trials or studies, as they are or with modifications. There is increasing recognition that this is not good practice as it is important to choose measures and make modifications with careful consideration of the content and utility of this measure in a new population and trial setting. Good practice recommendations are being developed; with the DIRUM database established as an open access repository for resource use measures which can be searched by disease, population and setting; (http://www.dirum.org/). However, this relies on health economists making their measures available and currently the database does not provide any critique of the strengths and limitations of the measures.

The importance of preparatory work to inform the resource use measure is increasingly recognised. This should not only focus on the development of a suitable measure but also consider approaches to value resource use to inform health economic analysis. Alongside this, is the need to ensure that the measure is sufficiently comprehensive, but still focused to capture the key items of interest (e.g. significant/main drivers of resource use and associated cost) to balance the trade-off between precision, effort, and burden to research participant). Thus, the REDUCE programme and the pre-programme work incorporates a systematic approach to the development of a suitable resource use measure for the REDUCE trial that can inform the health economic evaluation.

1.1. Summary of programme development work

Our pre-programme work enabled us to explore the following question: How should we assess the costs of REDUCE?

The objectives were to:

1) Identify the economic evidence of comparable psychosocial interventions on diabetic foot problems and any candidate resource use measure;
2) Gather information from the perspective of people with diabetic foot problems on the resources and associated costs and important economic outcomes;
3) Gather information from relevant stakeholders on important resources and outcomes for patients, the NHS and society;
4) Provide a descriptive synthesis from the data obtained to describe important and relevant resources, costs and outcomes to inform a future trial.

1.1.1. Methods

Health economics was a cross-cutting work package using data collected in a previous programme development grant. We undertook; a) a review of papers that reported any economic evaluations and also investigated whether there were any resource use measures that had been used in similar trials by searching the Database of Instruments for Resource Use Management (http://www.dirum.org/) and the NIHR library (https://www.journalslibrary.nihr.ac.uk/#/) and; b) conducted interviews with patients who were asked to comment on the important and relevant resources and costs associated with their diabetic foot problems. Similar questions were posed to healthcare professionals (HCPs) who also participated in interviews. The data collected were analysed to provide a summary of resources, costs and outcomes to be considered in a future trial.

1.1.2. Results

Objective 1: Review of the economic evidence
No suitable studies were identified that reported a full economic analysis based on the PICO. The DIRUM web-site failed to identify any comparable studies with the NIHR library identifying one trial (Jeffcoate *et al.,* 2017[1]) which reported the use of a patient diary to collect health resource use but a number of limitations were identified on the comprehensiveness of the measure.

Objective 2: Interviews with patients with a history of ulceration (n=20)
Patient interviews and question responses produced a content matrix of direct and indirect health and non-health care costs in describing their pathway of care. The following themes were identified in contextualising important resource use, cost drivers and outcomes for patients:

- ***Defining value of the REDUCE intervention:***
  Patients value the services they receive. The new service may provide added value but some patients did not view it as an alternative/replacement to current services.
- ***Getting treatment at the right time and place and how the new intervention would effect this:***
  Receiving appropriate care and reducing variation was seen as a key factor to patient's perception of any new services.
- ***Managing foot problems alongside other complex health needs:***
  Broader context of diabetes management versus diabetic foot problems needs to be taken into account including multiple services.

Objective 3: Interviews and questionnaires with health professionals (n=13)

Most professionals identified the importance of considering cost to the NHS, but also to the patient and family, with similar resource use identified to the patients. However, there was more emphasis on the impact on employment-related outcomes than the patients' responses. This identified the following themes in understanding the important resource use, cost drivers and outcomes:

- ***Skill mix:***
  The provision of different services and skill mix in delivering diabetic foot services being a key factor in contextualising the service across different sites.
- ***Follow-up:***
  The challenges of follow up care in managing the person with diabetic foot problems.
- ***Economic impact:***
  The gaps in terms of understanding the costs to the NHS, patient and society in treating diabetic foot problems.

Objective 4: Descriptive synthesis

Key findings from drawing together the above are:

- The importance of considering a perspective beyond the UK NHS/PSS should be considered in any future trial to include direct and indirect costs to the patient and family.
- A range of important and relevant resources have been described from the perspective of people affected by diabetic foot problems and clinicians which will enable us to develop a suitable resource use measure.
- Salient cost drivers should include all settings, with weighting given to primary care services. Both professionals and patients identified challenges in capturing this information given the recurrent 'revolving door' nature of managing diabetic foot problems.
- The findings did not go beyond identifying appropriate outcomes for use in an economic analysis other than clinical outcomes and in part, patient reported outcomes such as health-related quality of life. However, some of the findings from the patient and professional interviews point to the importance of looking beyond these as outcomes to include broader aspects.

1.1.3. Conclusions

We identified a paucity of current evidence on the cost-effectiveness of comparable interventions and that there is no suitable 'off the shelf' measure available. People with diabetic foot problems report a range of health and non-health care, direct and in-direct costs to be important. Further consideration is therefore needed to establish the most appropriate mechanism for reliable and valid capture of resource use data.

2. RATIONALE

2.1. Development of a resource use questionnaire to assess costs of DFU for the REDUCE trial

As part of the REDUCE programme, a key objective is to construct a resource use questionnaire (RUQ) for use in the REDUCE trial. This work has included: updating the literature since the

programme development grant to a) ensure no new measures have been developed and b) ensure the development of the RUQ is informed by good practice guidelines.

We have also conducted a review of the initial set of resource items derived from the programme development work and checked this for face validity with a panel of (n=6) patients and public representatives (5 people who have previously had a DFU and one family member and carer). This resulted in a content matrix of (n=40) items (Table 1). However, it is vital that a balance is made in capturing the most important drivers of resource use and cost against the precision required and burden associated in collecting and valuing resources. This preparatory work will enable us to form a judgement on how accurate (or precise) costs estimates need to be within a given study (Drummond *et al.,* 2005[2]).

To ensure this set of items represents a consensus view from the perspective of NHS professionals and people with DFUs, we will undertake a modified Delphi Study to identify which items are most important to capture the resources and associated costs of DFUs from the perspective of the NHS, patient and society. This will then be piloted in the REDUCE Pilot Trial to assess the viability of collecting the proposed outcome measures to inform the economic evaluation of the REDUCE trial. With the expectation that the pilot trial could (in principle) be treated as an internal (rather than external) pilot trial, we need to ensure that the RUQ is constructed to a format/content to enable the pilot data to contribute to the main economic evaluation.

3. OBJECTIVES AND OUTCOME MEASURES/ ENDPOINTS

3.1. Objectives

1. To agree a set of items for inclusion in a participant-reported measure to capture resource use for NHS, patient and society in DFU care.
2. To construct a participant-reported measure to capture resource use and costs for piloting as part of the REDUCE pilot trial.

4. STUDY DESIGN

Modified Delphi Survey compromising of up-to 3 rounds of on-line questionnaires to establish a consensus of resource items important to the NHS, patient and society in DFU care.

5. STUDY SETTING

The study setting is the UK. The participant population consists of two broad groups; patient and public representatives (including people who have had a DFU and carers and family members of people with DFUs) and healthcare professionals who work or previously have worked (e.g. now retired) with patients who have DFUs.

6. ELIGIBILITY CRITERIA

We will use broad eligibility criteria to 'mirror' the eligibility criteria for the REDUCE trial participants (e.g., adults who have had a previous DFU), and, in addition, include family members and carers of this group. For HCPs we will use broad criteria to capture the range of professions, grade and skill mix who would be involved in routine DFU care (e.g. diabetologists, podiatrists) and in delivering the REDUCE intervention (e.g. diabetes nurses/nurse educators).

We have taken a pragmatic approach and will use a convenience sampling approach to utilise existing (non-NHS) networks to recruit study participants. Whilst this may not capture a fully representative population, the Delphi Survey has been built on extensive qualitative work and literature reviews and we will have an opportunity to pilot the RUQ as part of the pilot trial described above.

6.1. Inclusion Criteria

Patient and public representatives:
- Age 18 or above.
- Has had a diabetic foot ulcer (DFU).
- Has sufficient command of the English language.

Carers and family members:
- Age 18 or above.
- A carer and/or family member of a person who has had a DFU.
- Has sufficient command of the English language.

Healthcare professionals:
- Age 18 or above.
- Involved in the routine care of patients with DFUs in the United Kingdom.

6.2. Exclusion Criteria

Patient and public representatives:
- Unwilling or unable to complete the electronic surveys.

Carers and family members:
- Unwilling or unable to complete the electronic surveys.

Healthcare professionals:
- Unwilling or unable to complete the electronic surveys.

7. STUDY PROCEDURES

7.1. Recruitment

7.1.1. Participant Identification

Patient and public representatives, carers and family members will be recruited from the existing REDUCE programme patient and public involvement group. The initial approach will be made by email from the REDUCE Programme Manager at the University of Nottingham who has ongoing access to the REDUCE PPI group contact details as part of the PPI co-ordination for the Programme Grant. Snowball sampling will be utilised where possible, for example, PPI members may wish to provide the study information to other family members not currently involved in the REDUCE PPI group. Other diabetes networks (e.g. regional diabetes networks via Diabetes UK) for people with diabetes may also be approached directly by the Chief Investigator and/or Programme Manager (whichever has access to the contact details as these will not be shared) to increase participant recruitment.

Healthcare professionals will be recruited from current networks and contacts known to the Chief Investigator and REDUCE researchers. This includes contacts from current existing networks (e.g. Diabetes UK, Diabetic Foot Networks), contacts who have registered their interest in REDUCE via separate email to the research team and/or Twitter, and the REDUCE programme management group. The initial approach to the PMG will be made by email from the REDUCE Programme Manager at the University of Nottingham who has ongoing access to the REDUCE programme management group contact details. The survey will also be promoted via the REDUCE_DFU Twitter account. Snowball sampling will be utilised where possible.

Potential participants will be provided with an initial invitation email (one for HCPs and another for PPI, carers and family members), which includes a link to Online Surveys (formerly Bristol Online Surveys, an electronic survey platform) where the participant information sheet, consent form and round 1 survey will be hosted.

7.2. Consent

Participants will provide consent to complete all questionnaires via the online survey platform after reading the participant information sheet and consent form. A copy of the participant information sheet and consent form wording will also be emailed to the participants. A record of consenting participants will be collated initially by Online Surveys. The contact details and consent will be downloaded by the Researchers at the University of Nottingham. The consent information will be added to the enrolment log and participant contact details will be stored in a separate spreadsheet. Both documents will be password-protected and stored on University of Nottingham servers, accessible by the Researchers on the delegation log.

7.3. Study Assessments

The modified Delphi survey will consist of up to three rounds of questionnaires. This number of questionnaire rounds has been consistently used within the literature to yield robust results (Stewart *et al.,* 2017[3]; Vogel *et al.,* 2019[4]). The Delphi method facilitates the gathering of a group consensus and further identifies areas where diversity of opinion exists (Hasson *et al.,* 2000[5]).

In line with the practice of previous electronically conducted Delphi studies, the link to the electronic questionnaires will be sent via email directly to participants and the data involved in each success round of the Delphi (e.g. distribution of panel responses and individual responses) will be distributed via the same method (Gill *et al.,* 2013[6]). It is estimated that each questionnaire will take approximately 30 minutes to complete.

7.3.1. First Round

Participants will receive the link to the electronic questionnaire and will be asked to rate each item within the questionnaire on a nine point Likert Scale according to the level of importance of the item. Within the scale, 1 will indicate 'not important and 9 will indicate 'very important'. Although it has been demonstrated that using a four point Likert scale generates stable findings in Delphi studies, a nine point scale will be used here as to facilitate the inclusion of a 'neither disagree or agree' and thus identify items that are either unclear or have neutral feelings towards them (Akins *et al.,* 2005[7]; Vogel *et al.,* 2019[4]; www.dirum.org/). Further space will be provided alongside each item to enable participants to comment and expand upon their thoughts in relation to the item and thus further inform the subsequent Delphi rounds (Stewart *et al.,* 2017[3]).

7.3.2. Second and Third Rounds

Participants will receive a covering email with the link to the Online Survey for the second round (and third round if applicable).
During the second round, participants will be provided with the items that reached consensus from the first round and an indication to the degree of consensus reached. Consensus will be considered to have been achieved if 75% agreement is reached (Diamond *et al.,* 2014[8]; www.dirum.org/). The second round questionnaire, a link to which will be present within the same email, will require participants to rate the items for which no consensus was reached in round one and rate any items that were added due to analysis of the free-text responses of participants from round one. The third round will proceed in the same manner. Given the resources available and further considerations in regards to participants (e.g. participant fatigue) no more than three rounds will be conducted if consensus is not reached upon the remaining items (Keeney *et al.,* 2006[9]).

A reminder to complete the relevant questionnaire will be emailed to each participant by the Researchers at the University of Nottingham if the questionnaire has not been completed after one week. A further reminder will be sent a week later (at two weeks).

7.4. Withdrawal Criteria

It will be assumed that participants no longer wish to take part in the study if explicitly stated in an email to the Researchers or if the second reminder does not result in the completion of a questionnaire. However, data provided by these individuals prior to their withdrawal will still be used in the study. Participants will be informed of this in the participant information sheet.

7.5. End of Study

The end of study will be defined as when the last participant has completed the final questionnaire. The CI will notify the Sponsor, participating sites and REC within 90 days of the end of study. The results will be analysed and the findings shared with the Programme Management Group within 3 months of the end of study.

8. SAFETY REPORTING

Adverse events are not anticipated for this low risk Delphi study and therefore will not be recorded.

9. DATA HANDLING

9.1. System and Compliance

Online Surveys will be used to host the questionnaires and the data. Online Surveys is GDPR compliant and ISO 27001 compliant (see: https://www.onlinesurveys.ac.uk/). Researchers at the University of Nottingham will access Online Surveys via username and password.

9.2. Source Data

Source data for this study consists of the online questionnaire data as collected via Online Surveys. This will include the records of consent and contact details.

9.3. Data Workflow, Access and Security

Only the Researchers at the University of Nottingham will have the details necessary to access Online Surveys where the survey data will initially be stored when returned by participants. Questionnaire data and personal details will be copied from Online Surveys and stored separately (questionnaire data will be attributable to study ID only) in a study dedicated restricted access folder on the University of Nottingham servers, accessible by UoN staff on the delegation log via UoN account usernames and passwords. Data held on the servers is encrypted and backed up every 24 hours. Participant IP addresses will not be captured. The anonymised data from each of the successive Delphi rounds will be shared with the Chief Investigator and Researchers at the University of Swansea, via a dedicated restricted access University of Nottingham cloud-based folder on Microsoft OneDrive/Teams. Contact details and the consent and enrolment log will be stored in a password-protected folder on University of Nottingham servers.

SCHE has access to a secure server at Swansea University, which is automatically backed up every 24 hours. Swansea University have Dell VxRail hyper converged stretched cluster and data is replicated between both campus sites. Virtual Servers are backed up daily to two Data Domains unit one at each campus and the data is replicated between them. Twice a month a tape backup is made and these are then taken offsite and stored in secure storage. The Dell VxRail cluster does not have encryption enable and backups are not encrypted, however no personal details will be stored on these servers. The SCHE folder on the servers is only accessible to designated SCHE staff and IT manager at College of Human and Health Sciences. All files will be password protected by the University of Nottingham and only shared with SCHE researchers on the delegation log.

SCHE will upload a copy of the final analysis set to the University of Nottingham cloud-based folder.

9.4. Archiving

At the end of the study, following completion of the end of study report, UHDB will securely archive all centrally held study related documentation for a minimum of 5 years. At the end of the defined archive period arrangements for confidential destruction will be made. It is the responsibility of each PI to ensure that data and all essential documents relating to the study are retained securely for a minimum of 5 years after the end of study, and in accordance with national legislation. UHDB will

notify sites when study documentation held at sites may be archived, and then destroyed. All archived documents must continue to be available for inspection by appropriate authorities upon request.

10. STATISTICS AND DATA ANALYSIS

10.1. Sample Size Calculation

A formal sample size calculation is not required for this study. A sample size of up to 50 participants, inclusive of people with DFUs, family members, carers and healthcare professionals will be sought for this phase of the study. The Delphi method is a widely accepted research approach; however, there is no consensus upon the size of the panel required. In general, a greater number of participants enhances the reliability of findings and reduces error (Keeney *et a.,* 2011[10]). However, Sekayi and Kennedy (2017[11]) noted that numbers greater than 20-30 become unwieldly in such an iterative process.

10.2. Statistical Analysis

The data yielded from the Modified Delphi will be analysed by the Researchers using SPSS. Descriptive statistical analysis will be performed including percentage of consensus upon each item, mean and standard deviation. A separate analysis plan will be produced for this study.

10.3. Procedure(s) to Account for Missing or Spurious Data

Due to the nature of online surveys, fields will be restricted to ensure responses are recorded for each item of the questionnaire. Missing data on individual items (if this occurs) will be noted.

11. MONITORING, AUDIT & INSPECTION

The Researchers must ensure that source documents and other documentation for this study are made available to study monitors, the REC or regulatory authority inspectors. Authorised representatives of the Sponsor may visit the participating sites to conduct audits/ inspections.

No formal monitoring of the trial will take place.

12. ETHICAL AND REGULATORY CONSIDERATIONS

12.1. Assessment and Management of Risk

Given the nature of this study the potential risks to those involved are relatively low as there will be no change to participants provided care.

12.2. Peer review

This study forms part of the REDUCE programme and was submitted to the funder as additional work as part of the programme. The internal research team have reviewed the study. The wider REDUCE programme has been peer reviewed as part of the NIHR Programme Grants for Applied Research application process.

12.3. Public and Patient Involvement

During the preliminary stages of project development, the involvement of members of the public including patients and carers was sought through the REDUCE patient and public involvement group. Further patient and public involvement will be maintained as the study progresses through engaging with the aforementioned group which meets face-to-face or via tele/video-conference three times per year (future meetings to be planned for July and November 2020). In addition, the REDUCE programme has an Independent Programme Steering Committee which has a dedicated patient and public representative.

12.4. Research Ethics Committee (REC) & Regulatory Considerations

The study will be conducted in compliance with the approved protocol and the Declaration of Helsinki. The protocol and all related documentation (e.g. informed consent form, participant information sheet, questionnaires) have been reviewed and received approval by a Research Ethics Committee (REC). The investigator will not begin any participant activities until approval from the HRA and REC has been obtained and documented. All documentation and correspondence must be retained in the study master file/study site file. Substantial amendments that require HRA and REC (where applicable) review will not be implemented until the HRA and REC grants a favourable opinion (with the exception of those necessary to reduce immediate risk to participants).

It is the responsibility of the CI to ensure that an annual progress report (APR) is submitted to the REC within 30 days of the anniversary date on which the favourable opinion was given, annually until the study is declared ended. The CI is also responsible for notifying the REC of the end of study (see Section 6.9) within 90 days. Within one year of the end of study, the CI will submit a final report with the results, including any publications/abstracts to the REC.

12.5. Protocol Compliance / Non-compliance Reporting

Every effort will be made to adhere to the protocol throughout the duration of the project. Any protocol deviation will be documented on a protocol deviation tracking log and reported to the Chief Investigator. Deviations from the protocol which are found to frequently reoccur will not be acceptable and thus will require immediate action (e.g. reported to Chief Investigator and Sponsor).

12.6. Notification of Serious Breaches to GCP and/or the Protocol

A “serious breach” is a departure from the protocol, Sponsor procedures (i.e. SOPs), or regulatory requirements which is likely to effect to a significant degree –

(a) The safety or physical or mental integrity of the subjects of the study; or
(b) The scientific value of the study.

If the Researcher is unsure if a non-compliance meets these criteria, they should consult the Sponsor for further guidance.

If a serious breach is identified the Chief Investigator will notify the Sponsor immediately (i.e. within 1 working day) using the 'Non-CTIMP Notification of a Serious Breach' form. The report will be reviewed by the Sponsor and CI, and where appropriate, the Sponsor will notify the REC within 7 calendar days of being made aware of the breach.

12.7. Data Protection and Participant Confidentiality

The study will be conducted in accordance with the Data Protection Act 2018. The investigator must ensure that participant's anonymity is maintained throughout the study and following completion of the study. Participants will be identified on all study specific documents (except for the enrolment log and contact details spreadsheet) only by the participants study specific identifier. The study identifier will consist of letters and numbers (for example, P01, FC01, H01, P02, etc. to denote the category and number of participants; Person with DFU, Family members/Carer, Healthcare Professional).This identifier will be recorded in the databases. The Study Site File at the University of Nottingham will hold the enrolment log detailing the study specific identifier alongside the names of all participants enrolled in the study, and a password-protected contact details spreadsheet.

All documents will be stored securely with access restricted to study staff and authorised personnel as listed on the delegation log. Participant contact details will only be accessible by the University of Nottingham Researchers.

Professor Fran Game will act as the custodian of the data generated in the study.

12.8. Financial and Other Competing Interests for the Chief Investigator, Principal Investigators at Each Site and Committee Members for the Overall Study Management

There are no anticipated financial or other competing interests.

12.9. Indemnity

As UHDB is acting as the research Sponsor for this study, NHS indemnity applies. NHS indemnity provides cover for legal liabilities where the NHS has a duty of care. Non-negligent harm is not covered by the NHS indemnity scheme. UHDB, therefore, cannot agree in advance to pay compensation in these circumstances. In exceptional circumstances an ex-gratia payment may be offered.

12.10. Amendments

If changes to the study are required these will be discussed with the Sponsor, who is responsible for deciding if an amendment is required and if it should be deemed substantial or non-substantial. Substantial amendments will be submitted to the relevant regulatory bodies (REC, HRA) for review and approval. The amendments will only be implemented after approval and a favourable opinion has been obtained. Non-substantial amendments will be submitted to the HRA for their approval/ acknowledgment. Amendments will not be implemented until all relevant approvals are in place.

12.11. Access to Final Study Dataset

Only the Chief Investigator and Researchers on the delegation logs at Swansea University and the University of Nottingham will have access to the final dataset.

13. DISSEMINATION POLICY

13.1. Dissemination Policy

This study will form part of the outputs for the REDUCE programme grant and we will present and publish findings in line with the REDUCE programme dissemination strategy. All presentations and publications will include the relevant current funding body acknowledgement. Prior to any publication or presentation (oral or written) a copy of the proposed publication or presentation will be provided to the funder at the same time as submission for publication or at least twenty-eight days before the date intended for publication (whichever is earlier).

13.2. Authorship Eligibility Guidelines and any Intended Use of Professional Writers

In principle, the main researcher(s) will assume the role as first/second author and other REDUCE project team members. Final authorship shall be in accordance with the International Committee of Journal Medical Editors (ICJME) guidance (http://www.icmje.org/recommendations/browse/roles-and-responsibilities/defining-the-role-of-authors-and-contributors.html) and will follow the authorship principles of the REDUCE programme.

14. REFERENCES

1. Jeffcoate W, Game F, Turtle-Savage V, Musgrove A, Price P, Tan W, et al. Evaluation of the effectiveness and cost-effectiveness of lightweight fibreglass heel casts in the management of ulcers of the heel in diabetes: a randomised controlled trial. *Health Technol Assess* 2017;21(34)

2. Drummond, MF, Sculpher, MJ, Torrance, GW, O'Brien, BJ & Stoddart, GL, 2005. Methods for the economic evaluation of health care programme. Third edition. Oxford: Oxford University Press.

3. Stewart D., Gibson-Smith K., MacLure K. Mair A., Alonso A., Codina C., et al. (2017) A modified Delphi study to determine the level of consensus across the European Union on the structures, processes and desired outcomes of the management of polypharmacy in older people. PLOS ONE https://doi.org/10.1371/journal.pone.0188348

4. Vogel C., Zwolinsky S., Griffiths C., (2019) A Delphi study to build consensus on the definition and use of big data in obesity research. International Journal of Obesity 43, 2573-2586

5. Hasson F, Keeney S, McKenna H. Research guidelines for the Delphi survey technique. *J Adv Nurs.* 2000 Oct;32(4):1008-15.

6. Gill FJ., Leslie G.D., Grech C., and Latour J.M., (2013) Using a web-based survey tool to undertake a Delphi study: application for nurse education research. Nurse Education Today vol.33, issue 11, pages 1322-1328

7. Akins RB, Tolson H, Cole BR. Stability of response characteristics of a Delphi panel: application of bootstrap data expansion. *BMC Med Res Methodol.* 2005 Dec 1;5:37. doi: 10.1186/1471-2288-5-37.

8. Diamond I.R., Grant R.C., Feldman B.M., Pencharz P.B., Ling S.C., Moore A.M. and Wales P.W., (2014) Defining consensus: A systematic review recommends methodologic criteria for reporting of Delphi studies. Journal of Clinical Epidemiology 67; 401-409

9. Keeney S, Hasson F, McKenna H. Consulting the oracle: ten lessons from using the Delphi technique in nursing research. *J Adv Nurs.* 2006 Jan;53(2):205-12.

10. Keeney S., Hasson F., and McKenna H. (2011) The Delphi Technique in Nursing and Health Research. Chichester, UK: Wiley DOI:10.1002/9781444392029

11. Sekayi, D., & Kennedy, A. Qualitative Delphi Method: A Four Round Process with a Worked Example. The Qualitative Report, 2017;22(10), 2755-2763.
